# Silent Sentinel: Incidental Discovery of Gunpowder-Induced Subconjunctival Foreign Bodies Three Years Post-firearm Injury

**DOI:** 10.7759/cureus.65514

**Published:** 2024-07-27

**Authors:** Anchal Tripathi, Mohini Agrawal, Atul Bhirud, Avinash Mishra

**Affiliations:** 1 Ophthalmology, Armed Forces Medical College, Pune, IND

**Keywords:** incidental discovery, subconjunctival foreign body, gunpowder foreign body, firearm injury, ocular trauma

## Abstract

Gunpowder-related ocular injuries are rare and can present significant diagnostic and management challenges. This case report describes a 27-year-old male who was discovered to have subconjunctival gunpowder foreign bodies during a routine annual medical examination. The patient presented with best-corrected visual acuity (BCVA) of 20/20 in both eyes and was asymptomatic, with no complaints of pain, visual disturbances, or other ocular symptoms.

Biomicroscopic examination revealed three gunpowder spherules embedded in the inferotemporal bulbar conjunctiva of the right eye, the largest measuring approximately 1.5 × 0.5 × 0.5 mm. When questioned, the patient recalled a firearm injury from 3 years prior but had never experienced any ocular discomfort or required treatment. Bilateral fundus examination and ultrasound B-scan confirmed the absence of intraocular foreign bodies and an intact scleral wall.

Given the patient’s asymptomatic presentation, no immediate treatment was recommended. Instead, the patient was advised to return for follow-up if symptoms developed. The case underscores the importance of thorough examination and patient history in determining the appropriate course of action for ocular foreign bodies, especially those related to gunpowder.

Despite their high-velocity impact, gunpowder-related foreign bodies are generally well tolerated, suggesting that observation can be a safe approach for asymptomatic cases. Nonetheless, a critical and patient-centered management strategy is essential, with interventions like phototherapeutic keratectomy (PTK) reserved for cases where foreign bodies cause visual impairment or other complications. This case report highlights the need for individualized treatment plans and suggests that observation can be a viable approach when dealing with asymptomatic ocular foreign bodies.

## Introduction

Sub-conjunctival foreign bodies are pretty uncommon in occurrence as compared to superficial conjunctival and intracorneal foreign bodies [1]. There have been very few case reports on subconjunctival foreign bodies [1-3]. Moreover, gunpowder injuries leading to ocular foreign bodies are extremely rare [4]. Gunpowder is a mixture of nitrocellulose and nitroglycerin, which releases high-pressure gases when combusted [4]. Nitrocellulose, the primary constituent of gunpowder, breaks down into nitrates and carbon, both of which are generally non-toxic [4]. Therefore, the removal of asymptomatic gunpowder foreign bodies may not be necessary [5-8]. This case report explores an unusual instance of incidentally detected asymptomatic gunpowder-related ocular foreign bodies, highlighting the need for careful examination and tailored management strategies.

## Case presentation

**Figure 1:**
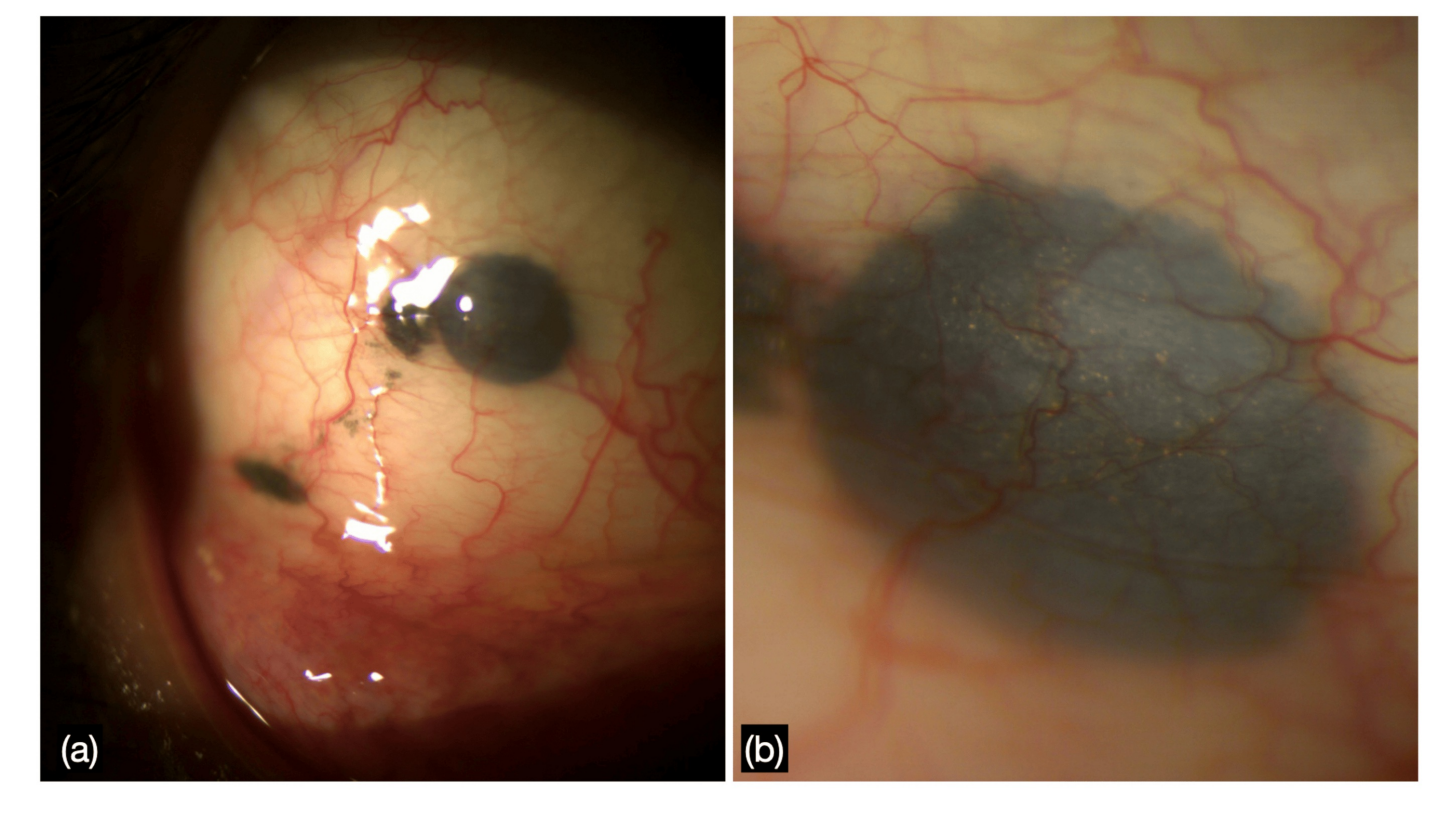
Biomicroscopic examination The examination revealed subconjunctival gunpowder foreign bodies in the inferotemporal bulbar conjunctiva, appearing as three spherules adjacent to each other, with the largest measuring approximately 2.3 × 1.5 × 0.5 mm; with vascularization over the superior surface.

A 27-year-old male presented to the ophthalmology outpatient department for a routine annual medical examination. His best-corrected visual acuity (BCVA) was 20/20 in both eyes. Biomicroscopic examination of the right eye revealed subconjunctival gunpowder foreign bodies in the inferotemporal bulbar conjunctiva, appearing as three spherules adjacent to each other, with the largest measuring approximately 2.3 x 1.5 x 0.5 mm; with vascularization over the superior surface (Figure 1). On further inquiry, the patient recalled a firearm injury 3 years prior but denied any symptoms or previous treatment in his right eye. He was asymptomatic at the time of examination. The patient was a military personnel by occupation and suffered the injury during an operation, via a smoothbore barreled shotgun. He had suffered a closed globe injury in his right eye leading to gunpowder traces along with conjunctival laceration during that time, which was sutured. The black lesion was diagnosed as gunpowder based on its typical shiny appearance and the history given by the patient.

The patient’s bilateral fundus evaluation was within normal limits, and an ultrasound B-scan of the right eye showed no intraocular foreign bodies, with an intact scleral wall. Given the asymptomatic nature of the foreign bodies, no treatment was offered. Close follow-up was recommended to monitor for any changes in symptoms or ocular health. The patient remained asymptomatic during the follow-up period of 2 years, with no progression of ocular findings or development of symptoms related to the subconjunctival foreign bodies. Regular surveillance was planned to ensure the stability of the ocular condition and to intervene promptly, if necessary.

## Discussion

Gunpowder injuries can result in not only metallic foreign bodies but also non-metallic gunpowder particles [4]. While metallic foreign bodies typically require removal to avoid rust rings and other complications, gunpowder particles have shown a high level of tolerance within the eye [4-7]. This patient exhibited no signs of inflammation or irritation, suggesting that gunpowder-related foreign bodies, in the subconjunctival areas, might not necessitate immediate removal, if asymptomatic [5-7].

Several reports suggest observation as a viable treatment option for asymptomatic patients with ocular foreign bodies [5-7]. However, penetrating keratoplasty, phototherapeutic keratectomy (PTK), and other interventions might be required in cases of intracorneal foreign bodies, where there is significant visual impairment or discomfort [9,10]. For this case, conservative management was chosen due to the lack of symptoms and the patient’s stability. To the best of our knowledge, no such case has been reported previously.

## Conclusions

Gunpowder-related ocular injuries can be well tolerated, but careful evaluation and follow-up are essential to ensure patient safety. The case illustrates that not all ocular foreign bodies require immediate removal, especially if the patient is asymptomatic. To our knowledge, no such case has been reported in the past. Experience and critical judgment are crucial in managing such cases, with observation being a safe approach in many instances. However, new techniques like PTK provide viable options for cases with intracorneal foreign bodies, underscoring the need for individualized treatment plans, although the literature itself is not very clear where to surgically intervene.
